# Evaluation of Potential Predictive Biomarkers for Defining Brain Radiotherapy Efficacy in Non-Small Cell Lung Cancer Patients with Brain Metastases: A Case Report and a Narrative Review

**DOI:** 10.3390/clinpract13060136

**Published:** 2023-11-30

**Authors:** Angelo Luciano, Luigi Liguori, Giovanna Polcaro, Francesco Sabbatino, Stefano Pepe

**Affiliations:** 1 Oncology Unit, Department of Medicine, Surgery and Dentistry, University of Salerno, 84081 Baronissi, Italy; angelo.luciano2@unina.it (A.L.); luigi.liguori2@unina.it (L.L.); gpolcaro@unisa.it (G.P.); spepe@unisa.it (S.P.); 2 Oncology Unit, Department of Clinical Medicine and Surgery, University of Naples “Federico II”, 80131 Naples, Italy

**Keywords:** brain metastases, NSCLC, predictive biomarkers for radiotherapy, radiotherapy, prognostic scores, WBRT, BRAF mutation

## Abstract

Non-small cell lung cancer (NSCLC) is the second most common cancer worldwide, resulting in 1.8 million deaths per year. Most patients are diagnosed with a metastatic disease. Brain metastases are one of the most common metastatic sites and are associated with severe neurological symptoms, shorter survival, and the worst clinical outcomes. Brain radiotherapy and systemic oncological therapies are currently used for controlling both cancer progression and neurological symptoms. Brain radiotherapy includes stereotactic brain ablative radiotherapy (SBRT) or whole brain radiotherapy (WBRT). SBRT is applied for single or multiple (up to ten) small (diameter less than 4 cm) lesions, whereas WBRT is usually applied for multiple (more than ten) and large (diameter greater than 4 cm) brain metastases. In both cases, radiotherapy application may be viewed as an overtreatment which causes severe toxicities without achieving a significant clinical benefit. Thus far, a number of scoring systems to define the potential clinical benefits derived from brain radiotherapy have been proposed. However, most are not well established in clinical practice. In this article, we present a clinical case of a patient with advanced NSCLC carrying a BRAF^V600E^ mutation and brain metastases. We review the variables in addition to applicable scoring systems considered to have potential for predicting clinical outcomes and benefits of brain radiotherapy in patients with advanced NSCLC and brain metastases. Lastly, we highlight the unmet need of specific scoring systems for advanced NSCLC patients with brain metastases carrying oncogene alterations including BRAF^V600E^ mutations.

## 1. Introduction

Non-small cell lung cancer (NSCLC) is the second most common cancer worldwide, resulting in 2 million diagnoses and 1.8 million deaths per year [1]. The most significant risk factor for NSCLC is cigarette smoke due to its carcinogenic chemicals [2]. This risk increases with the number of cigarettes smoked per day in addition to the number of years spent smoking. Other well-known risk factors include asbestos, radon, and silica exposure [2].

There are various histologic subtypes of NSCLC including squamous cell carcinoma (SCC), adenocarcinoma (ADC), adeno-squamous carcinoma (ASC), large-cell carcinoma (LCC), and NSCLC not otherwise specified (NOS) [3]. In addition, types of NSCLC are also classified as oncogene or non-oncogene addicted, based on the presence/absence of specific tumor alterations [4]. The former includes tumors carrying tumor alterations such as Epidermal Growth Factor Receptor (EGFR) (10–15%), Anaplastic Lymphoma Kinase (ALK) (3–7%), and V-Raf Murine Sarcoma Viral Oncogene Homolog B (BRAF) (2–4%) [4].

Treatment of NSCLC includes surgery, chemotherapy, targeted therapy, immunotherapy, and radiotherapy. Surgery with tumor resection represents the primary treatment for stage I and II NSCLC, whereas for the stage III disease it is an important component of the multimodality approach in association with radiotherapy and chemotherapy [2]. Chemotherapy can include the combination of platinum derivatives (cisplatin or carboplatin) with other cytotoxic agents such as gemcitabine, paclitaxel, pemetrexed, nab-paclitaxel, and vinorelbine as well as the use of single chemotherapeutic agents both in early and advanced stages of the disease [2]. Targeted therapy with tyrosine kinase inhibitors (TKIs) in addition to other types of small molecules is only applicable to small subset of NSCLC patients carrying oncogene alterations. To date, numerous targeted therapies have been integrated into clinical practice for many types of oncogene alterations. The most notable include EGFR inhibitors (i.e., afatinib and osimertinib) for EGRF mutations, ALK inhibitors (i.e., alectinib and brigatinib) for ALK translocations, and BRAF inhibitor (dabrafenib) for BRAF mutations [2].

Immunotherapy with immune checkpoint inhibitors (ICIs) such as anti-programmed cell death-1 (PD-1) (cemiplimab, nivolumab and pembrolizumab), anti-programmed death-ligand 1 (PD-L1) (atezolizumab and durvalumab), and anti-cytotoxic T-lymphocyte antigen-4 (CTLA-4) (ipilimumab and tremelimumab) is revolutionizing the treatment landscape of several types of cancer including non-oncogene addicted NSCLC. This novel immune-therapeutic approach is currently utilized as a single agent or in combination with chemotherapy in both early and advanced stage of the disease [5].

Lastly, radiotherapy is currently used either with a radical intent, in combination with chemotherapy for treatment of primary tumors, or as a single agent with palliative intent, for treatment of bone or brain metastases [2]. The latter represent a major site of the metastatic disease [6] and are the consequence of a complex process that includes induction of angiogenesis, malignant cell blood dissemination, extravasation, proliferation, and survival [7]. Brain radiotherapy is administrated either as stereotactic ablative radiotherapy (SBRT) or as whole brain radiotherapy (WBRT), based on the patient and tumor characteristics [8]. SBRT delivers a high dose to limited sized targets, which represents a reasonable strategy for patients who are not candidates for neurosurgery in the presence of single or multiple (up to ten) small (diameter less than 4 cm) brain metastases. However, WBRT is the best choice in cases of multiple (more than ten) and large (diameter greater than 4 cm) brain metastases [8]. In both cases, radiotherapy is utilized both to relieve the neurological symptoms and to inhibit the tumor progression, but its limited efficacy and derived neurotoxicity can lead to the selection of the best supportive care option as a valid alternative [9]. Hence, there is a need to define potential biomarkers which can assist with identifying patients with the greatest benefits to gain from brain radiotherapy, and those who could avoid treatments with little or no benefit. Thus far, a number of scoring systems have been proposed [10,11,12].

Here, by presenting the clinical outcomes obtained from WBRT in a patient with brain metastases from an advanced NSCLC carrying a BRAF^V600E^ mutation, we analyzed the potential variables as well as the available scoring systems to predict clinical outcomes and benefits of brain radiotherapy in patients with NSCLC and brain metastases.

## 2. Case Presentation

**Figure 1:**
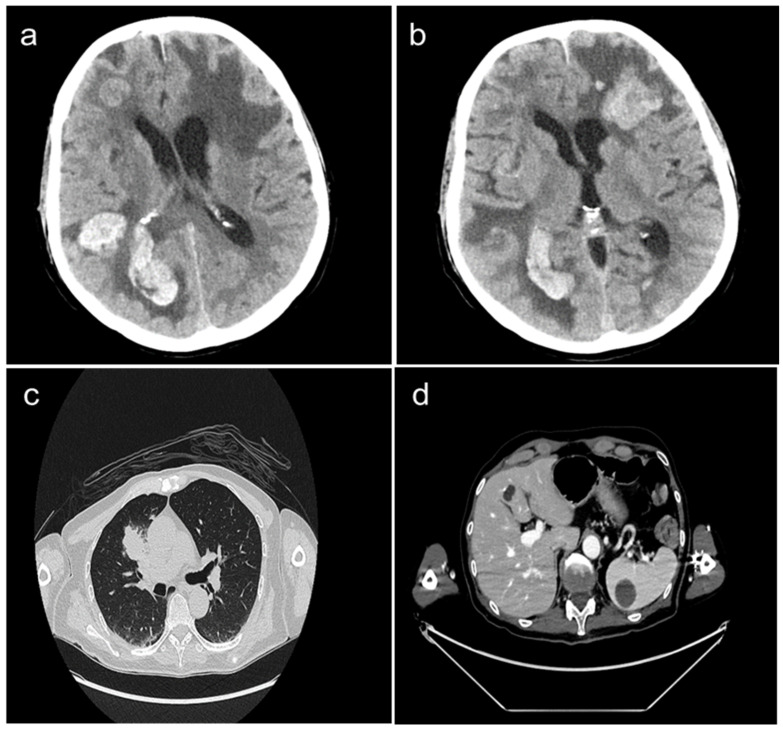
Whole body CT-scan performed at diagnosis showing the presence of multiple brain metastases localized in the right frontoparietal, occipital (panel **a**), and left frontal lobes (panel **b**). Large mass in the right-upper lung lobe (panel **c**), liver metastasis (10 mm), and spleen metastasis (30 mm) (panel **d**) are also presented.

A 62-year-old Caucasian male, non-smoker, visited the first aid facility of our University Hospital, presenting with dyspnea, visual impairments, and dizziness. His neurological syndrome deteriorated within a few hours. A radiological evaluation with a computed tomography (CT) scan demonstrated the presence of multiple brain metastases (of which one had a diameter greater than 4 cm) localized in the left frontal, right frontoparietal, and occipital lobes as well as in the right cerebellar hemisphere. Massive edema, compression of cerebellum, right lateral ventricle, and subfalcine herniation were also described (Figure 1a,b). Other tumor localizations included the presence of a large mass in the right-upper lung lobe and multiple lymph nodal, liver (10 mm), and spleen (30 mm) metastases (Figure 1c,d).

Basal tumoral markers were in normal range, with the except of neuron-specific enolase (NSE) (14.9 ng/mL). The baseline electrocardiogram (ECG) showed sinus rhythm at 82 beats per minute (bpm) and a QT corrected for heart rate (QTc) of 425 milliseconds (ms). Blood pressure displayed 125/80 mm of mercury (mmHg) and the saturation of peripheral oxygen (SpO_2_) was 98%. According to brain metastasis localization, the patient had pyramidal syndrome, numbness, ocular ptosis, spastic paraplegia and aphasia, neurocognitive decline, and loss of self-care. An analysis of biohumoral parameters demonstrated a significant increase in lactate dehydrogenase (LDH), aspartate aminotransferase (AST), alanine aminotransferase (ALT), iron, ferritin, bilirubin (especially non-direct index), blood urea, and glycemic levels, whereas those of albumin, transferrin, sodium, potassium, and calcium were reduced. Blood count was normal. The Eastern Cooperative Oncology Group Performance Status (ECOG PS) was recorded as 3. The Karnofsky performance status (KPS) was 40%. Supportive care was started immediately with the administration of dexamethasone 8 mg every 8 h, mannitol 18% every 6 h, and levetiracetam 500 mg bid. After 4 days of treatment with supportive care, the patient’s neurological symptomatology benefited little and a percutaneous CT-assisted lung biopsy was performed. After 7 days, tumor histopathological analysis confirmed the diagnosis of lung adenocarcinoma with a PD-L1 tumor proportion score (TPS) between 1 and 49%. Moreover, to further define the number, the dimension, and the characteristics of the brain metastases, a brain magnetic resonance imaging (MRI) scan was proposed. However, the patient refused the brain MRI scan due to his claustrophobia. Therefore, based on the limited benefit to the neurological symptoms, the general clinical conditions of the patient, and the presence of one large brain metastasis (a diameter of more than 4 cm) which rules out the application of SBRT, in accordance with our multidisciplinary group (which included radiologists, molecular biologists, neuroradiologists, neurosurgeons, and radiotherapists) we decided to apply WBRT without the results of the brain MRI scan. WBRT with hippocampal inclusion was immediately started (30 Gray in 10 fractions). During the 7 days following the completion of radiotherapy, molecular analysis of the tumor biopsy demonstrated the presence of BRAF^V600E^ mutation. Based on this result, the patient was a candidate for the BRAF and MEK inhibitor combination of dabrafenib and trametinib. However, at the same time, the patient’s neurological symptoms deteriorated with the development of pyramidal syndrome, ocular ptosis, spastic paraplegia, aphasia, neurocognitive decline, and an inability to swallow. As a result, the treatment with dabrafenib and trametinib was not started. A comparison between the present biohumoral parameters and those during pre-radiotherapy treatment demonstrated a decrease in hemoglobin (HGB) levels, red blood cells (RBC), and platelet (PLT) count (11.4 g/dL vs. 14.1 g/dL for HGB; 7.42 × 10^6^/µL vs. 6.02 × 10^6^/µL for RBC and 282 × 10^3^/µL vs. 161 × 10^3^/µL for PLT), whereas white blood cells (WBC), neutrophil (NEU) count, and NEU-to-lymphocyte ratio (NLR) were increased (25.3 × 10^3^/µL vs. 17.14 × 10^3^/µL for WBC, 24.14 × 10^3^/µL vs. 16.02 × 10^3^/µL for NEU and 34 vs. 54 for NLR). Cardiac evaluation showed a progressive elevation of heart rate (maximum value of 179 bpm), atrial flutter development, and alterations in ST trait. Tumoral markers were higher than basal (Ca 125 was 49.9 U/mL vs. 26.6 U/mL and Ca 19.9 was 36.6 U/mL vs. 19 U/mL). Unfortunately, after more 6 days, despite the specific cardiologic treatment, clinical conditions deteriorated further and patient died.

## 3. Discussion

### 3.1. The Role of WBRT in Advanced NSCLC Patients with Brain Metastases

SBRT and WBRT play a major role in the treatment of brain metastases. WBRT represents the best choice of treatment in cases of multiple (more than ten) and large (diameter greater than 4 cm) brain metastases, regardless of the tumor type. In NSCLC, WBRT has been shown to improve both neurological symptoms and disease control [8]. Nevertheless, WBRT is also associated with temporary (short-term) or persistent (long-term) toxicity [9]. The former includes alopecia, dermatitis, fatigue, otitis, nausea, and alterations in both memory and executive functions [13]. Persistent toxicity includes impaired physiological function of the hippocampus, ataxia, insomnia, dysphasia, and dementia [13]. Short and long-term WBRT toxicity can be reduced by the exclusion of selective brain areas such as the hippocampus, leading to an improvement of neurocognitive function, functional autonomy, and quality of life [13]. In the case we have described, the patient would not have obtained any long-term benefit from hippocampal exclusion due to his very poor prognosis. In addition, since the limited clinical benefit obtained by supportive care alone, our main aim was to maximize the short-term benefit gained from the control of neurological symptoms by utilizing WBRT. As a result, we decided to include the hippocampus area. Unfortunately, the patient did not achieve any clinical benefit from WBRT despite the inclusion of the hippocampus area.

### 3.2. The Potential Role of BRAF Mutations and ICI-Based Immunotherapy in Advanced NSCLC Patients with Brain Metastases

Brain metastases of our patient were derived from a NSCLC carrying a BRAF^V600E^ mutation. In this type of tumor, the administration of BRAF and MEK inhibitors has been demonstrated to improve both overall survival and response rate, even in the presence of brain metastases [14]. However, to the best of our knowledge, no clinical study has evaluated the intracranial efficacy of the BRAF and MEK inhibitor combination in BRAF^V600E^ NSCLC patients with multiple symptomatic brain metastases. In contrast, several preclinical and clinical studies have been investigating the combination of the BRAF inhibitor and radiotherapy as well as of BRAF and MEK inhibitors in melanoma patients carrying similar alterations in BRAF, even in presence of multiple brain metastases [15]. It has been shown that aberrant activation of the RAS/BRAF pathway in melanoma cells increases the resistance to radiations whereas its inhibition restores the radio-sensitization of cancer cells [16]. In addition, Sambade et al. have demonstrated a synergistic effect of BRAF inhibitor and radiation in melanoma cell death through an increase in the G1 arrest of cancer cells, laying the basis for combinatorial therapeutic approaches [17]. Unfortunately, even in melanoma patients, the combination of the BRAF inhibitor and radiation has been limited by a significant increase in severe toxicities [18]. As a result, BRAF inhibitors are administered either before or after WBRT. However, several lines of evidence have demonstrated a relevant clinical benefit obtained by BRAF and MEK inhibitors in melanoma patients carrying BRAF^V600E^ [19]. In this setting, the BRAF and MEK inhibitor combination induces 68% of the intracranial disease control rate (stable disease, partial response, and complete response of 37%, 26%, and 5%, respectively) [19]. Further studies are required to validate the efficacy of the combination of BRAF and MEK inhibitors in NSCLC patients carrying the BRAF^V600E^ mutation with brain metastases. Unfortunately, since the rapid worsening of the clinical condition of our patient, we were unable to propose this novel type of treatment. However, we suggest that oncogene alterations should be further investigated as factors which could potentially impact the process for selecting the best treatment option for brain metastases in advanced NSCLC patients carrying oncogene alterations.

In addition to BRAF and MEK inhibitors, in our case, the combination of chemotherapy and anti-PD-1-based immunotherapy could represent an alternative therapeutic approach. In the definition of the best therapeutic algorithm, one should take into account that in addition to oncogene alterations, PD-L1 tumor expression plays a significant role in the choice of treatment for advanced NSCLC patients [5]. Moreover, in the patient we have described, according to PD-L1 tumor expression, platinum-based chemotherapy and anti-PD-1-based immunotherapy could have represented an alternative therapeutic option. This therapeutic approach has clearly been demonstrated to improve both the response rate and survival outcomes of treated patients as compared to standard platinum-based chemotherapy [5]. However, the implementation of ICI-based immunotherapy for the treatment of advanced NSCLC patients with brain metastases has raised several questions including (i) the need of biomarkers to predict the clinical efficacy of treated patients; (ii) the potential crossing of blood–brain barrier by monoclonal antibodies (mAbs) targeting PD-1 or PD-L1 to demonstrate their efficacy in the challenging setting of patients with brain metastases; and (iii) the potential side effects on brain functions from ICIs.

Among the biomarkers with the ability to predict the clinical benefit of NSCLC patients treated with ICIs, PD-L1 expression is the most validated in the literature. Indeed, patients with a higher PD-L1 expression have a higher likelihood of achieving a clinical benefit from ICIs. However, some evidence in the literature also demonstrated the discordance of the PD-L1 expression between the primary site tumor and metastatic brain tumors. This discordance occurs in more than 20% of patients with advanced NSCLC and brain metastases, with the greatest discordance for the PD-L1 expression subgroup ranging between 1% and 49% [20]. This discordance is relevant and potentially affects the prediction about the efficacy of the ICI-based immunotherapy. Unfortunately, in our case report, the very poor clinical conditions of the patient and his severe neurological symptoms did not allow us to perform this type of evaluation.

The blood–brain barrier represents the main issue limiting the efficacy of oncological drugs in the treatment of primary or secondary brain tumors. Indeed, this is present in some drugs, such as the mAbs, since their molecular weight and structure have the intrinsic difficulty to crossing this barrier. As a result, one might wonder whether ICIs cross the blood–brain barrier and what is their real efficacy in brain metastases. Although ICIs are mainly mAbs and consequently cannot reach intracranial tumors. Several clinical trials have also clearly demonstrated their efficacy in cancer patients with brain metastases. These results may be explained by the cytotoxic effect of T cells activated by ICIs in the extracranial site tumors which are also able to cross the blood–brain barrier and destroy intracranial tumor cells [21]. Further studies are required to clarify this mechanism as well as to develop novel strategies to increase the efficacy of ICIs for the treatment of brain metastases.

ICIs are a well-known cause of a wide spectrum of immune-related adverse events (irAEs). Despite this, neurological irAEs are much less frequent than others such as gastrointestinal, skin, or endocrine toxicities, with a prevalence of less than 5% of treated patients, though their severe or even fatal consequences are markedly feared [22]. Neurological irAEs for the most part involve the peripheral nervous system and can cause peripheral neuropathies, myasthenia gravis, myositis, and Guillain-Barrè syndrome. In addition, they can also impair brain functions with a wide range of syndromes including encephalitis, central nervous system vasculitis, non-specific meningitis, transverse myelitis, multiple sclerosis-like, demyelinating syndromes, and cranial neuropathies. The cornerstone of the management of these irAEs include two main aspects: the discontinuation of the ICI (temporarily or permanently) and the administration of immunosuppressive treatment. Unfortunately, in patients with brain metastases the diagnosis of these syndromes is often complicated by the potential overlap of the symptomatology between irAEs and brain metastases [22].

However, thus far, no study has performed a comparison to determine which is the most effective therapeutic approach in NSCLC patients and no study testing sequential strategies such as the BRAF and MEK inhibitor combination as compared to the combination of immunotherapy and chemotherapy in NSCLC patients carrying BRAF^V600E^ is available. In BRAF^V600E^ melanoma patients, two recent clinical studies have shown that the presence of the BRAF^V600E^ mutation may influence the best therapeutic sequence in advanced melanoma. Specifically, a major benefit is achieved from an up-front immunotherapy as compared to an up-front BRAF and MEK inhibitor combination [23,24]. In the clinical case we have described, the administration of chemotherapy and anti-PD-1-based immunotherapy was limited by (i) the availability of clinical data in a setting with patients carrying BRAF^V600E^ with symptomatic brain metastases; (ii) the detrimental effect of high dose steroids on the efficacy of anti-PD-1 therapy; (iii) the clinical conditions of the patient (PS ECOG 3, KPS 3); and (iv) the Italian guideline indications which limit the administration of platinum-based chemotherapy and anti-PD-1 therapy following the failure of a prior BRAF and MEK inhibitor combination. One could expect that based on the faster activity the combination of BRAF and MEK inhibitors should represent the best therapeutic option in this specific subgroup of patients. Further prospective studies are needed to clarify this specific aspect.

In our case, we did not promptly start the BRAF and MEK inhibitor combination as the tumor oncogene analysis was still pending. We promptly started WBRT immediately following histological tumor analysis because of the neurological symptoms. As a result, we were unable to assess the potential tumor–brain response or the clinical benefits deriving from the sequential strategies of targeting agents and radiotherapy. Nevertheless, WBRT alone did not provide any clinical benefit, severe toxicities were developed and the BRAF and MEK inhibitors were not administrated. Based on the obtained results, one might suppose that the best supportive care option could be a valid alternative to WBRT.

### 3.3. The Role of the Scoring Systems to Predict the Benefit of WBRT in Advanced NSCLC Patients with Brain Metastases

Thus far, a number of scoring systems for predicting the clinical outcomes from WBRT in patients with brain metastases have been proposed. They include the Radiation Therapy Oncology Group–Recursive Partitioning Analysis (RTOG-RPA) and the WBRT-30-NSCLC scores [11,25]. The RTOG-RPA score is a statistical methodology which creates a regression tree according to the prognostic significance. For its validation, both pre-treatment and treatment-related variables were analyzed [25] (Table 1).

Among all the prognostic variables identified (Table 2), three RPA classes were defined.

The first class included patients who had a KPS ≥ 70, age < 65 years, and no extracranial disease. The second included patients with a KPS ≥ 70 and at least one unfavorable prognostic factor. The last group included patients with a KPS < 70. According to this score, an increased survival from WBRT for brain metastases was obtained only by patients in the first class [25]. In contrast, no benefit was achieved in patients with a KPS ≤ 70 and higher tumor burden.

The second score system, the WBRT-30-NSCLC score, was developed for patients with intracerebral metastases from NSCLC. Eight factors were investigated in NSCLC patients receiving WBRT including age, gender, KPS, interval from diagnosis of NSCLC to WBRT, pre-WBRT systemic treatment, primary tumor control, number of intracerebral metastases, and metastasis outside the brain (Table 3) [11]. Among the variables analyzed, age, KPS, systemic treatment, and metastasis outside the brain were found to correlate with 6-month patient survival.

Then for each identified prognostic variable a score was assigned (Table 4) and 4 groups of patients were identified with 6-month survival rates of 3, 26, 65, and 100% [11].

It was proposed for patients with a score of 9–10 points to be treated with a short-course of WBRT because their chance of survival was poor, whereas NSCLC patients with a score of 17–18 points should receive a long-course of WBRT, due to their increased chance of surviving longer [11]. When we compare these scoring systems, the WBRT-30-NSCLC score appears to be more accurate for NSCLC as it differentiates the patients with intracerebral metastases from the NSCLC patients who will die within 6 months or survive longer. However, both scoring systems display some limitations. Firstly, neither is able to distinguish between WBRT with hippocampal inclusion from that with hippocampal exclusion. Secondly, both scoring systems are strongly influenced by KPS and do not consider the biological and molecular features of tumors in addition to the evaluation of biohumoral parameters of the NSCLC patients. The exclusion of hippocampal area from the radiotherapy field has a lower impact on neurological declines and preserves memory and concentration. Alternatively, the evaluation of biohumoral parameters can help to identify patients with a short lifespan. In our case, we did not apply any of the scoring systems available. An analysis of the class risk score by both scoring systems shows a poor risk class for both RTOG-RPA and WBRT-30-NSCLC. Indeed, our patient’s results positioned them in the third class according to RTOG-RPA and a score of 11 points according to WBRT-30-NSCLC. Ultimately, although the patient was affected from a BRAF^V600E^ NSCLC, with multiple sites of metastasis (brain, spleen, and liver), both scoring systems were efficient in predicting no clinical benefit from WBRT. In our case, it is likely that both scoring systems performed well because they are both strongly influenced by the poor KPS of the patient. The other items included in WBRT-30-NSCLC do not help to better stratify the prognosis or the prediction of the clinical benefit in our case when compared to RTOG-RPA. However, no scoring systems are available for this type of patient or for other types of oncogene addicted tumors, thus far. Further studies are required to grant personalized radiotherapy treatment for this patient population. In our case, in addition to the clinical features analyzed in both scoring systems, we also detected a progressive heart failure, an elevation of tumoral markers, a lowering of serum hemoglobin levels, an increasing of platelet count, and a worsening of liver and renal function. These parameters should also be considered since they may help to identify an imminent exitus of the patient and therefore no benefit from WBRT. Among these parameters, NLR might be one of the best candidates as a prognostic and/or predictive factor. Indeed, two studies have already demonstrated that high values for both pre-treatment and post-treatment NLR predict a poor chance of survival in NSCLC patients’ brain metastases treated with SBRT or WBRT [26,27]. In our patient both pre-treatment and post-treatment NLRs were extremely high and correlated with poor prognosis and displayed no benefits from WBRT. However, to validate the prognostic/predictive role of these parameters including NLR, further studies are needed within the specific subset of NSCLC patients with brain metastases carrying the BRAF^V600E^ mutation treated with WBRT.

## 4. Conclusions

Beyond the value of the specific parameters, our case report seems to suggest that a rapid worsening of these parameters may be the best factor to predict which patients have the worst prognosis. In these conditions, in addition to poor risk classes from RTOG-RPA and WBRT-30-NSCLC scores, best supportive care should lead a valid alternative option of selecting the best supportive care in order to avoid any useless treatment. Finally, with this work we want to highlight the unmet need of a validated scoring systems to better predict the prognosis and the potential benefit from WBRT in advanced NSCLC patients with brain metastases carrying BRAF^V600E^ in addition to other oncogene alterations. We believe that novel scoring systems should also integrate more biohumoral and clinical parameters in order to better identify patients with a short lifespan whom can avoid WBRT.

## Figures and Tables

**Table 1:** List of pre-treatment and treatment-related variables analyzed for the identification of the RTOG-RPA scoring system.

Variable	Description
Brain metastases	Alone
	With other brain metastases
Primary lesion	Controlled
	Uncontrolled
Primary lesion site	Lung
	Breast
	Other
Histology	Squamous
	Adenocarcinoma
	Large cell
	Small cell
	Melanoma
	NSC
	Other
Prior brain surgery	None
	Yes
Time interval from diagnosis of primary to brain metastases	≤2 years
	>2 years
Headache	Absent
	Present
Seizure	Absent
	Present
Visual disturbance	Absent
	Present
Neurologic function	None
	Minor
	Moderate
	Major
Midline shift	No
	Yes
Mass effect	No
	Yes
Location of lesions	Frontal
	Temporal
	Parietal
	Occipital
	Basal ganglia/thalamus
	Cerebellum
	Brainstem
Sentinel location of lesions	Frontal
	Temporal
	Parietal
	Occipital
	Basal ganglia/thalamus
	Cerebellum
	Brainstem
Sentinel lesion side	Left
	Right
	Midline
Necrotic center	No
	Yes
Number of lesions	Single
	Multiple
Tumor response	Complete
	Partial
	Stable
	Progression
KPS	30–40
	50–60
	70–80
	90–100
Area (mm^2^)	0–400
	401–900
	901–1600
	>1601
Age (years)	<40
	40–44
	45–49
	50–54
	55–59
	60–64
	65–69
	>70
Total dose (cGy)	2400–3499
	3500–4000
	4001–5279
	5280–6079
	6080–6719
	6720–9000

Abbreviations: cGy: centigray; KPS: Karnofsky performance status; and NSC: non-small-cell.

**Table 2:** Univariate analysis of pre-treatment and treatment-related variables tested for the identification of the RTOG-RPA scoring system.

Variable	Comparison	*p*-Value
Brain metastases	alone vs. with other metastases	<0.0001
KPS	≥70 vs. <70	<0.0001
Age (years)	<65 vs. ≥65	<0.0001
Prior surgery	no vs. yes	0.005
Histology	squamous vs. small cell vs. others	<0.0001
Primary lesion	controlled vs. uncontrolled	<0.0001
Primary site	breast vs. lung and others	0.001
Time interval	<2 years vs. >2 years	0.004
Number of lesions	single vs. multiple	0.021
Sentinel lesion side	left and/or right vs. midline	0.038
Sentinel location	frontal, temporal, parietal, occipital, and basal ganglia/thalamus vs. cerebellum and brainstem	0.033
Neurologic function	no vs. yes	<0.0001
Headache	no vs. yes	0.003
Total dose (cGy)	≥5200 vs. <5200	<0.0001
Tumor response	complete or partial vs. stable or progressive	0.019

Abbreviations: cGy: centigray; and KPS: Karnofsky performance status.

**Table 3:** List of variables analyzed for the identification of the WBRT-30-NSCLC scoring system.

Variable	Description
Age (years)	≤62
	≥63
Gender	Male
	Female
KPS	<70
	70
	>70
Interval from diagnosis of NSCLC to WBRT	≤1 months
	≥2 months
Pre-WBRT systemic treatment	No
	Yes
Control of the primary tumor	No
	Yes
Number of intracerebral metastases	1–3
	≥4
Metastasis outside the brain	No
	Yes

Abbreviations: KPS: Karnofsky performance status; NSCLC: Non-small-cell lung cancer; and WBRT: whole brain radiotherapy.

**Table 4:** WBRT-30-NSCLC score.

Variable	Factor Score
Age (years)	
≤62	4
≥63	2
KPS	
<70	1
70	3
>70	5
Pre-WBRT systemic treatment	
No	2
Yes	4
Number of intracerebral metastases	
1–3	4
≥4	2
Metastasis outside the brain	
No	5
Yes	2

Abbreviations: KPS: Karnofsky performance status; and WBRT: whole brain radiotherapy.

## Data Availability

The data presented in this study are available on request from the corresponding author.
